# Supplementary material for: Identification of developmental disorders including autism spectrum disorder using salivary miRNAs in children from Bosnia and Herzegovina
Source: PLoS One. 2020 Apr 30;15(4):e0232351. doi: 10.1371/journal.pone.0232351 (PMC7192422; doi:10.1371/journal.pone.0232351)
Supplement: S1 Table — (DOCX) [file pone.0232351.s001.docx]

**S1 Table.** Percentage of obtained Ct values for every cohort and for each individual miRNA

| miRNA | miR-191-5p | miR-7-5p | miR- 23a-3p | miR- 27a-3p | miR-28-5p | miR-30e-5p | miR-32-5p | miR-127-3p | miR-140-3p | miR- 218-5p | miR- 335-3p | miR- 3529-5p | miR- 628-5p | miR-2467-5p | Total |
| --- | --- | --- | --- | --- | --- | --- | --- | --- | --- | --- | --- | --- | --- | --- | --- |
| Obtained results for TD | 88% | 92% | 84% | 84% | 80% | 84% | 76% | 84% | 84% | 80% | 84% | 92% | 84% | 80% | 84% |
| Obtained results for ASD | 82% | 71,7% | 74,3% | 76,9% | 58,9% | 74,3% | 46,1% | 48,7% | 71,7% | 64,1% | 69,2% | 79,4% | 64,1% | 51,2% | 66,6% |
| Obtained results for DD | 87,5% | 75% | 75% | 87,5% | 68,75% | 87,5% | 68,75% | 68,75% | 81,25% | 87,5% | 93,7% | 87,5% | 81,2% | 68,7% | 79,9% |
| Total obtained results for a miRNA | 85% | 78,7% | 77,5% | 81,2% | 67,5% | 80% | 60% | 63,7% | 77,5% | 73,7% | 78,7% | 85% | 73,7% | 63,7% | 74,7% |
