# Supplementary material for: Identification of developmental disorders including autism spectrum disorder using salivary miRNAs in children from Bosnia and Herzegovina
Source: PLoS One. 2020 Apr 30;15(4):e0232351. doi: 10.1371/journal.pone.0232351 (PMC7192422; doi:10.1371/journal.pone.0232351)
Supplement: S2 Table — (DOCX) [file pone.0232351.s002.docx]

**S2 Table.** Shown is the detailed logistic regression performance of individual miRNAs between TD and DD cohorts.

| Statistic | -2 Log(Likelihood) (Probability) | Wald  (Probability) | Specificity  (Validation) | Sensitivity  (Validation) | Accuracy  (Validation) | ROC |
| --- | --- | --- | --- | --- | --- | --- |
| miR-191-5p | 0.426 (0.513) | 0.426 (0.513) | 0% (0%) | 100% (100%) | 68.18% (60%) | 0.510 |
| miR-7-5p | 3.646 (0.056) | 3.146 (0.076) | 4.55% (0%) | 100% (100%) | 68.18% (70%) | 0.642 |
| miR-23a-3p | 9.507 (0.002) | 8.040 (0.005) | 36.36% (0%) | 88.64% (85.71%) | 71.21% (60%) | 0.746 |
| miR-27a-3p | 3.454 (0.063) | 3.196 (0.074) | 20.83% (0%) | 90.48% (88.89%) | 65.15% (80%) | 0.602 |
| miR-28-5p | 2.298 (0.130) | 2.229 (0.135) | 9.09% (0%) | 95.45% (85.71%) | 66.67% (60%) | 0.598 |
| miR-30e-5p | 0.046 (0.830) | 0.046 (0.831) | 0% (0%) | 100% (100%) | 66.67% (70%) | 0.560 |
| miR-32-5p | 11.208 (0.001) | 9.500 (0.002) | 36.36% (33.33%) | 84.09% (85.71%) | 68.18% (70%) | 0.765 |
| miR-127-3p | 0.007 (0.933) | 0.007 (0.933) | 0% (0%) | 100% (100%) | 65.15% (80%) | 0.523 |
| miR-140-3p | 1.912 (0.167) | 1.896 (0.169) | 8.70% (50%) | 95.35% (75%) | 65.15% (70%) | 0.617 |
| miR-218-5p | 1.052 (0.305) | 1.050 (0.306) | 8.33% (0%) | 97.62% (88.89%) | 65.15% (80%) | 0.591 |
| miR-335-3p | 0.473 (0.492) | 0.459 (0.498) | 0% (0%) | 100% (100%) | 68.18% (60%) | 0.588 |
| miR-3529-3p | 0.008 (0.928) | 0.008 (0.928) | 0% (0%) | 100% (100%) | 66.67% (70%) | 0.530 |
| miR-628-5p | 6.247 (0.012) | 5.492 (0.019) | 17.39% (0%) | 86.05% (75%) | 62.12% (60%) | 0.696 |
| miR-2467-5p | 0.046 (0.829) | 0.046 (0.830) | 0% (0%) | 100% (100%) | 69.70% (50%) | 0.510 |
