# Supplementary material for: Identification of developmental disorders including autism spectrum disorder using salivary miRNAs in children from Bosnia and Herzegovina
Source: PLoS One. 2020 Apr 30;15(4):e0232351. doi: 10.1371/journal.pone.0232351 (PMC7192422; doi:10.1371/journal.pone.0232351)
Supplement: S3 Table — (DOCX) [file pone.0232351.s003.docx]

**S3 Table.** Shown is the detailed logistic regression performance of individual miRNAs between TD and ASD cohorts.

| Statistic | -2 Log(Likelihood) (Probability) | Wald  (Probability) | Specificity  (Validation) | Sensitivity  (Validation) | Accuracy  (Validation) | ROC |
| --- | --- | --- | --- | --- | --- | --- |
| miR-191-5p | 0.018 (0.892) | 0.018 (0.892) | 0% (0%) | 100% (100%) | 58.82% (60%) | 0.533 |
| miR-7-5p | 3.739 (0.053) | 3.310 (0.069) | 47.62% (25%) | 93.33% (83.33%) | 74.51% (60%) | 0.675 |
| miR-23a-3p | 5.406 (0.020) | 4.778 (0.029) | 52.17% (100%) | 78.57% (87.5%) | 66.67% (90%) | 0.702 |
| miR-27a-3p | 2.189 (0.139) | 2.039 (0.153) | 30.43% (0%) | 78.57% (100%) | 56.86% (80%) | 0.575 |
| miR-28-5p | 1.340 (0.247) | 1.301 (0.254) | 14.29% (50%) | 86.67% (100%) | 56.86% (80%) | 0.602 |
| miR-30e-5p | 0.509 (0.476) | 0.505 (0.477) | 14.29% (0%) | 96.67% (100%) | 62.75% (60%) | 0.506 |
| miR-32-5p | 7.363 (0.007) | 6.388 (0.011) | 50.00% (40%) | 80.65% (100%) | 68.63% (70%) | 0.753 |
| miR-127-3p | 0.341 (0.559) | 0.340 (0.560) | 13.64% (0%) | 93.10% (85.71%) | 58.82% (60%) | 0.538 |
| miR-140-3p | 2.013 (0.156) | 1.939 (0.164) | 31.82% (33.33%) | 79.31% (85.71%) | 58.82% (70%) | 0.657 |
| miR-218-5p | 2.968 (0.085) | 2.795 (0.095) | 25.00% (0%) | 90.32% (60%) | 64.71% (30%) | 0.653 |
| miR-335-3p | 0.089 (0.765) | 0.089 (0.765) | 0% (0%) | 100% (100%) | 56.86% (70%) | 0.483 |
| miR-3529-3p | 0.112 (0.738) | 0.110 (0.740) | 0% (0%) | 100% (100%) | 62.75% (40%) | 0.564 |
| miR-628-5p | 1.890 (0.169) | 1.813 (0.178) | 28.57% (25%) | 83.33% (83.33%) | 60.78% (60%) | 0.619 |
| miR-2467-5p | 0.125 (0.723) | 0.125 (0.724) | 16.67% (0%) | 92.59% (77.78%) | 56.86% (70%) | 0.539 |
