# Supplementary material for: Identification of developmental disorders including autism spectrum disorder using salivary miRNAs in children from Bosnia and Herzegovina
Source: PLoS One. 2020 Apr 30;15(4):e0232351. doi: 10.1371/journal.pone.0232351 (PMC7192422; doi:10.1371/journal.pone.0232351)
Supplement: S4 Table — (DOCX) [file pone.0232351.s004.docx]

**S4 Table.** Shown is the detailed logistic regression performance of individual miRNAs between TD and non-ASD DD cohorts.

| Statistic | -2 Log(Likelihood) (Probability) | Wald  (Probability) | Specificity  (Validation) | Sensitivity  (Validation) | Accuracy  (Validation) | ROC |
| --- | --- | --- | --- | --- | --- | --- |
| miR-191-5p | 0.480 (0.488) | 0.462 (0.497) | 100% (100%) | 0% (0%) | 62.07% (70%) | 0.566 |
| miR-7-5p | 1.280 (0.258) | 1.230 (0.267) | 100% (100%) | 20% (0%) | 72.41% (60%) | 0.558 |
| miR-23a-3p | 3.127 (0.077) | 2.557 (0.110) | 100% (100%) | 14.29% (0%) | 79.31% (30%) | 0.701 |
| miR-27a-3p | 0.932 (0.334) | 0.858 (0.354) | 94.44% (100%) | 0% (0%) | 58.62% (70%) | 0.540 |
| miR-28-5p | 0.656 (0.418) | 0.661 (0.416) | 100% (100%) | 0% (0%) | 72.41% (40%) | 0.571 |
| miR-30e-5p | 0.022 (0.881) | 0.022 (0.882) | 100% (100%) | 0% (0%) | 62.07% (70%) | 0.495 |
| miR-32-5p | 9.301 (0.002) | 5.905 (0.015) | 76.47% (100%) | 66.67% (0%) | 72.41% (80%) | 0.828 |
| miR-127-3p | 0.315 (0.575) | 0.313 (0576) | 100% (100%) | 0% (0%) | 62.07% (70%) | 0.581 |
| miR-140-3p | 0.509 (0.476) | 0.466 (0.495) | 100% (100%) | 0% (0%) | 72.41% (40%) | 0.577 |
| miR-218-5p | 0.767 (0.381) | 0.671 (0.413) | 100% (100%) | 0% (0%) | 72.41% (40%) | 0.649 |
| miR-335-3p | 0.024 (0.878) | 0.024 (0.877) | 100% (100%) | 0% (0%) | 65.52% (60%) | 0.553 |
| miR-3529-3p | 2.915 (0.088) | 2.443 (0.118) | 89.47% (50%) | 20% (0%) | 65.52% (30%) | 0.689 |
| miR-628-5p | 0.099 (0.754) | 0.098 (0.754) | 100% (100%) | 0% (0%) | 72.41% (40%) | 0.542 |
| miR-2467-5p | 0.001 (0.975) | 0.001 (0.975) | 100% (100%) | 0% (0%) | 65.52% (60%) | 0.553 |
