# Supplementary material for: Identification of developmental disorders including autism spectrum disorder using salivary miRNAs in children from Bosnia and Herzegovina
Source: PLoS One. 2020 Apr 30;15(4):e0232351. doi: 10.1371/journal.pone.0232351 (PMC7192422; doi:10.1371/journal.pone.0232351)
Supplement: S5 Table — (DOCX) [file pone.0232351.s005.docx]

**S5** **Table.** Shown is the detailed logistic regression performance of individual miRNAs between ASD and non-ASD DD cohorts.

| Statistic | -2 Log(Likelihood) (Probability) | Wald  (Probability) | Specificity  (Validation) | Sensitivity  (Validation) | Accuracy  (Validation) | ROC |
| --- | --- | --- | --- | --- | --- | --- |
| miR-191-5p | 0.031 (0.861) | 0.031 (0.861) | 0% (0%) | 100% (100%) | 75.56% (40%) | 0.460 |
| miR-7-5p | 0.039 (0.844) | 0.039 (0.843) | 0% (0%) | 100% (100%) | 71.11% (80%) | 0.478 |
| miR-23a-3p | 0.126 (0.722) | 0.128 (0.720) | 0% (0%) | 100% (100%) | 71.11% (80%) | 0.567 |
| miR-27a-3p | 0.037 (0.847) | 0.037 (0.847) | 0% (0%) | 100% (100%) | 71.11% (80%) | 0.486 |
| miR-28-5p | 1.112 (0.292) | 1.117 (0.291) | 7.69% (0%) | 96.88% (75%) | 71.11% (60%) | 0.632 |
| miR-30e-5p | 0.605 (0.437) | 0.608 (0.436) | 0% (0%) | 100% (100%) | 75.56% (40%) | 0.521 |
| miR-32-5p | 0.161 (0.689) | 0.157 (0.692) | 0% (0%) | 100% (100%) | 68.89% (70%) | 0.576 |
| miR-127-3p | 1.273 (0.259) | 1.262 (0.261) | 0% (0%) | 100% (100%) | 71.11% (80%) | 0.601 |
| miR-140-3p | 0.407 (0.523) | 0.413 (0.520) | 0% (0%) | 100% (100%) | 71.11% (80%) | 0.599 |
| miR-218-5p | 0.071 (0.791) | 0.069 (0.793) | 0% (0%) | 100% (100%) | 73.33% (60%) | 0.530 |
| miR-335-3p | 0.397 (0529) | 0.396 (0529) | 0% (0%) | 100% (100%) | 71.11% (80%) | 0.541 |
| miR-3529-3p | 0.271 (0.603) | 0.260 (0.610) | 0% (0%) | 100% (100%) | 71.11% (80%) | 0.538 |
| miR-628-5p | 0.121 (0.728) | 0.122 (0.727) | 0% (0%) | 100% (100%) | 73.33% (60%) | 0.553 |
| miR-2467-5p | 0.001 (0.981) | 0.001 (0.981) | 0% (0%) | 100% (100%) | 68.89 (70%) | 0.442 |
