# Supplementary material for: Identification of developmental disorders including autism spectrum disorder using salivary miRNAs in children from Bosnia and Herzegovina
Source: PLoS One. 2020 Apr 30;15(4):e0232351. doi: 10.1371/journal.pone.0232351 (PMC7192422; doi:10.1371/journal.pone.0232351)
Supplement: S6 Table — (DOCX) [file pone.0232351.s006.docx]

**S6 Table.** Details on performance of the PLS-DA prediction models on the analysed cohorts.

| Cohorts Analysed | Sensitivity | Specificity | Accuracy |
| --- | --- | --- | --- |
| TD – DD | 36% | 86.27% | 69.74% |
| TD – ASD | 64% | 88.57% | 78.33% |
| TD – non-ASD DD | 88% | 64.29% | 79.49% |
| ASD – non-ASD DD | 18,75 | 94.29% | 70.59% |
|  | |  |  |
