# Supplementary material for: Identification of developmental disorders including autism spectrum disorder using salivary miRNAs in children from Bosnia and Herzegovina
Source: PLoS One. 2020 Apr 30;15(4):e0232351. doi: 10.1371/journal.pone.0232351 (PMC7192422; doi:10.1371/journal.pone.0232351)
Supplement: S7 Table — (DOCX) [file pone.0232351.s007.docx]

**S7 Table.** MiRNAs with Variable Importance in Projection (VIP) value over 1.0 for each cohort within the PLS-DA.

| TD – DD | TD – ASD | TD – non-ASD DD | ASD – non-ASD DD |
| --- | --- | --- | --- |
| miR-23a-3p (1.996) | miR-23a-3p (2.091) | miR-32-5p (2.203) | miR-3529-3p (1.896) |
| miR-7-5p (1.468) | miR-32-5p (1.519) | miR-23a-3p (1.969) | miR-28-5p (1.394) |
| miR-32-5p (1.460) | miR-7-5p (1.358) | miR-628-5p (1.241) | miR-23a-3p (1.332) |
| miR-27a-3p (1.249) | miR-27a-3p (1.2) | miR-7-5p (1.238) | miR-140-3p (1.249) |
| miR-628-5p (1.135) | miR-628-5p (1.145) |  | miR-628-5p (1.209) |
| miR-140-3p (1.107) | miR-140-3p (1.135) |  | miR-191-5p (1.058) |
| miR-2467-5p (1.01) |  |  |  |
|  | |  |  |
