# Supplementary material for: Identification of developmental disorders including autism spectrum disorder using salivary miRNAs in children from Bosnia and Herzegovina
Source: PLoS One. 2020 Apr 30;15(4):e0232351. doi: 10.1371/journal.pone.0232351 (PMC7192422; doi:10.1371/journal.pone.0232351)
Supplement: S8 Table — (DOCX) [file pone.0232351.s008.docx]

**S8 Table.** Power analysis (where the number of final sample sizes for each miRNA or test can be seen) on logistic regression which have shown statistically significant ability in differentiating between cohorts

| Logistic Regression Power analysis | | | |
| --- | --- | --- | --- |
| Groups | ASD – TD | TD – DD | TD – non-ASD DD |
| Sample Size | 61,000 | 76,000 | 39,000 |
| Odds Ratio | 80,000 | 84,000 | 34,500 |
| x parm pi | 0,409 | 0,320 | 0,360 |
| R2 other | 0,000 | 0,000 | 0,000 |
| alpha err prob | 0,050 | 0,050 | 0,050 |
| Power | 0,935 | 0,965 | 0,983 |
