# Supplementary material for: Identification of developmental disorders including autism spectrum disorder using salivary miRNAs in children from Bosnia and Herzegovina
Source: PLoS One. 2020 Apr 30;15(4):e0232351. doi: 10.1371/journal.pone.0232351 (PMC7192422; doi:10.1371/journal.pone.0232351)
Supplement: S9 Table — (DOCX) [file pone.0232351.s009.docx]

**S9 Table.** Power analysis on Mann-Whitney U test on TD – DD cohorts

| Mann-Whitney U test Power analysis (TD – DD) | | | | | | |
| --- | --- | --- | --- | --- | --- | --- |
| miRNA | miR-23a-3p | miR-32-5p | miR-7-5p | miR-628-5p | miR-140-3p | miR-2467-5p |
| Sample Size Group 1 | 21 | 19 | 23 | 21 | 21 | 20 |
| Sample Size Group 2 | 41 | 29 | 40 | 38 | 41 | 31 |
| Effect Size | 1,469 | 1,339 | 0,499 | 1,166 | 0,796 | 0,627 |
| Power | 0,999 | 0,991 | 0,45 | 0,984 | 0,814 | 0,565 |
