# Supplementary material for: Identification of developmental disorders including autism spectrum disorder using salivary miRNAs in children from Bosnia and Herzegovina
Source: PLoS One. 2020 Apr 30;15(4):e0232351. doi: 10.1371/journal.pone.0232351 (PMC7192422; doi:10.1371/journal.pone.0232351)
Supplement: S10 Table — (DOCX) [file pone.0232351.s010.docx]

**S10 Table.** Power analysis on Mann-Whitney U test on TD – ASD cohorts

| Mann-Whitney U test Power analysis (TD – ASD) | | | | | |
| --- | --- | --- | --- | --- | --- |
| miRNA | miR-23a-3p | miR-32-5p | miR-7-5p | miR-628-5p | miR-140-3p |
| Sample Size Group 1 | 21 | 19 | 23 | 21 | 21 |
| Sample Size Group 2 | 29 | 18 | 28 | 25 | 28 |
| Effect Size | 1,516 | 1,492 | 0,647 | 1,243 | 0,813 |
| Power | 0,999 | 0,99 | 0,595 | 0,979 | 0,768 |
