# Supplementary material for: Identification of developmental disorders including autism spectrum disorder using salivary miRNAs in children from Bosnia and Herzegovina
Source: PLoS One. 2020 Apr 30;15(4):e0232351. doi: 10.1371/journal.pone.0232351 (PMC7192422; doi:10.1371/journal.pone.0232351)
Supplement: S11 Table — (DOCX) [file pone.0232351.s011.docx]

**S11 Table.** Power analysis on Mann-Whitney U test on TD – non-ASD DD cohorts

| Mann-Whitney U test Power analysis (TD – non-ASD DD) | | | | |
| --- | --- | --- | --- | --- |
| miRNA | miR-23a-3p | miR-32-5p | miR-628-5p | miR-2467-5p |
| Sample Size Group 1 | 21 | 19 | 21 | 20 |
| Sample Size Group 2 | 12 | 11 | 13 | 11 |
| Effect Size | 1,358 | 1,134 | 1,010 | 1,245 |
| Power | 0,943 | 0,804 | 0,773 | 0,884 |
