# Supplementary material for: Identification of developmental disorders including autism spectrum disorder using salivary miRNAs in children from Bosnia and Herzegovina
Source: PLoS One. 2020 Apr 30;15(4):e0232351. doi: 10.1371/journal.pone.0232351 (PMC7192422; doi:10.1371/journal.pone.0232351)
Supplement: Attachment — Submitted filename: PLOS One Review 113019.docx [file pone.0232351.s013.docx]

Overall, this is an interesting manuscript which interrogates an emerging biomarker family in a clinical disorder with a great need for an objective, biologic diagnostic aid.

The novelty of the manuscript lies in its unique ethno-geographic cohort, and the potential to confirm previously-published, non-invasive biomarkers in that cohort. The authors have done a thorough job providing clinical characteristics of the participants, and the statistical approach appears sound.

My main concern is regarding the interpretation of the results, and the way the discussion frames them (in the context of the existing literature). The authors are missing a key citation, which involves the largest study of saliva microRNA in children with autism (PMID: 30926572). This study showed that saliva microRNA alone does not provide sufficient accuracy for differentiating children with ASD from peers with DD or TD. This is why additional studies (e.g. citation 54) have moved to poly-omic analysis of both microRNA and other human and microbial RNAs. For this reason, a study of <100 children is not really a validation study - especially since the authors have used different saliva collection kits, different RNA extraction techniques, and different RNA quantification protocols. The discussion does not include any mention of these factors. There is also no discussion of the study's limitations.

Here are a few additional specific observations/suggestions:

The intro would benefit from a discussion of the advantages to saliva relative to blood collection.

This study does not show that microRNAs can be used as early as birth for ASD diagnosis. There are studies to show this type of test is not even a priority for parents of children with ASD (PMID: 30903561).

I worry that the sample size (n=25 for controls) does not support separate training/test sets. A power analysis would be useful here.

Table 1 needs to compare sex/age across groups (were there differences?). Also, details about diet, oral hygiene, and oral meds would help with interpretation of the findings.

Were any participants excluded for inability to expectorate?

Please provide data on RNA quality and concentrations. Box plots for miRNA expression across groups is necessary.

Were all miRNAs detectable? In what % of participants? What were the Ct values?

Line 381-2: I would suggest a thorough discussion of why the results might differ across saliva microRNA studies: sex, age, race, ethnicity, saliva collection devices, RNA extraction devices, RNA quant approaches, composition of ASD/DD groups, severity of ASD group, small sample size of the current study (relative to newer studies in the field) could all be factors that led to differences across studies.

If I am interpreting the text correctly, 47% of children with ASD received a clinical evaluation. This is a limitation that should be discussed.
