# Supplementary material for: Identification of developmental disorders including autism spectrum disorder using salivary miRNAs in children from Bosnia and Herzegovina
Source: PLoS One. 2020 Apr 30;15(4):e0232351. doi: 10.1371/journal.pone.0232351 (PMC7192422; doi:10.1371/journal.pone.0232351)
Supplement: Attachment — Submitted filename: Response to Reviewers.docx [file pone.0232351.s014.docx]

Dear Reviewers,

Thank you so much for your time and such thorough comments and suggestions. All of your suggestions and comments have been addressed and the manuscript has been updated accordingly. Answers to your comments and how they have been addressed, in detail, please find below.

Once more thank you.

Sincerely,

Authors

REVIEWER 1

1. **It is unclear how the 14 miRNAs were selected. It seems to be based on reference 49 (A comparative review of microRNA expression patterns in autism spectrum disorder. Frontiers in psychiatry. 2016); however, I do not see much overlap with the 27 miRNAs discussed in that paper.**

We have selected the 14 miRNAs based on their performance in Hicks et al., 2016.

2. **The validation set” was mentioned, but it is unclear what that is.**

Our validation set was made up of 10% of randomly selected samples from the overall analysed cohort.

REVIEWER 2

**1.	The authors are missing a key citation, which involves the largest study of saliva microRNA in children with autism (PMID: 30926572).**

As suggested we have included the key citation PMID: 30926572 within the discussion section (line: 100, 460).

**2.	This is why additional studies (e.g. citation 54) have moved to poly-omic analysis of both microRNA and other human and microbial RNAs. For this reason, a study of <100 children is not really a validation study - especially since the authors have used different saliva collection kits, different RNA extraction techniques, and different RNA quantification protocols. The discussion does not include any mention of these factors. There is also no discussion of the study's limitations.**

Poliomic analysis has been addressed and included in discussion. We agree that this is not a validation study, it is an additional test for assessment of children with developmental disorders and have made contextual changes accordingly (line: 37)

Study limitations have been addressed, (line: 425)

**3.	The intro would benefit from a discussion of the advantages to saliva relative to blood collection.**

As suggested the advantages of salivary relative to blood collection were discussed in the introduction (line: 94)

**4.	This study does not show that microRNAs can be used as early as birth for ASD diagnosis. There are studies to show this type of test is not even a priority for parents of children with ASD (PMID: 30903561).**

Based on the suggestion we have adjusted the utility of miRNAs as diagnostic tools as early as birth.

**5.	I worry that the sample size (n=25 for controls) does not support separate training/test sets. A power analysis would be useful here.**

Power analysis has been performed and is included within the Supplementary Material 2.

**6.	Table 1 needs to compare sex/age across groups (were there differences?). Also, details about diet, oral hygiene, and oral meds would help with interpretation of the findings.**

We have adjusted table 1 according to the suggestions for the control group and we have added the suggested information (sex/age) regarding DD children in Table 2. We do not have information regarding diet, oral hygiene and oral meds.

**7.	Were any participants excluded for inability to expectorate**

There was one sample mentioned in Materials and Methods section (line: 150)

**8.	Please provide data on RNA quality and concentrations. Box plots for miRNA expression across groups is necessary.**

Data on RNA quality and concentrations is provided to the reviewer attached as an excel file (“Samples ASD”) and the box plots for miRNA expression across groups has been added as a figure in the manuscript (Figure 1).

**9.	Were all miRNAs detectable? In what % of participants? What were the Ct values?**

Data regarding the percentages of detected miRNAs have been added and can be found in Supplementary file 1. Raw Ct value data has been attached as an excel file (“Ct value and normalization”)

**10. Line 381-2: I would suggest a thorough discussion of why the results might differ across saliva microRNA studies: sex, age, race, ethnicity, saliva collection devices, RNA extraction devices, RNA quant approaches, composition of ASD/DD groups, severity of ASD group, small sample size of the current study (relative to newer studies in the field) could all be factors that led to differences across studies.**

According the suggestion we have addressed the mentioned differences in the discussion (427)

**11. If I am interpreting the text correctly, 47% of children with ASD received a clinical evaluation. This is a limitation that should be discussed.**

The mentioned limitation has been addressed in the discussion (line: 452)

REVIEWER 3

**1.	Overall, the paper is well written, but would be strengthened with further presentation of the data in the paper.**

As suggested by the reviewer, additional data was added in the paper through Figure 1, Table 3 and Supplementary Tables 1 to 11 a and 2.

**2.	While all results are displayed in the text it is difficult to gauge which are common and how** **great changes are by comparing sections of text. Unfortunately, the naming of miRNA does not lend itself well to easy distinction at a glance. The paper would be aided by separating DE miRNA into tables that include directional fold change differences and p-values. I realize this adds cost of redundancy, but that is offset by the value of the clarity. Tables for DE results and then for prediction results of both single logistic and PLS-DA results would be useful. I believe a heatmap showing the specific DE miRNA across all the samples and labelled with the category would be useful to see how consistent the results are across the cohort. As it stands, the buildup to the lone figure is somewhat lost, but important to understand the strength of the marker set used and the diversity of the sample cohort.**

The naming of miRNAs was addressed. Differentially expressed miRNAs with directional fold changes and p-values were included in Table 3. Regarding the suggested tables for individual miRNA LR performance and PLS-DA performance we have decided that it would be more suitable to add such tables as supplements to text in order to preserve the consistency of data presented in the text.

After constructing boxplots proposed by Reviewer 2 and heatmaps by Reviewer 3 for visualizing the expression patterns of individual miRNAs within studies groups, we have decided that boxplots visually offer more clarity and information.

Table showing differential expression is added

**3.	There seems to also be some clarification needed in certain areas. For instance, it was unclear why only 26 children have clinical opinion from the child psychologist and initial thoughts were that the rest were deemed non-ASD or non-DD. It is clarified that only a subset of these children were evaluated, but the message was that of agreement between CARS-II and the clinical assessment. Perhaps the table can call that section Concordance between Child Psychiatrist and CARS-II or have that added to be more clear?**

Clinical evaluation and the mentioned issues by the reviewer were thoroughly addressed in materials and methods and discussion sections of the manuscript. Concordance between child psychiatrist and CARS 2 has been added in Table 2.

4. **It is not clear how samples were separated into training and validation. There is a line at the end of the results section that mentions 5 samples in the validation being too small for meaningful conclusions. Is that the number for all or just the non-ASD DD vs TD? The number in each set should be provided to understand the design and relevance of the training and validation sets**.

The used program randomly separates the training and validation sets, multiple iterations were performed, and the results did not differ significantly. A smaller validation subset was selected only for non-ASD DD because their sample size was smaller than others. Based on your suggestion the whole process of validation has been more thoroughly explained within the materials and methods section of the manuscript.

5. **Please ensure all miRNA names are miR- and not miRNA-. Also watch for some uses of mir**-.

Based on your suggestion the naming of miRNAs has been addressed.

**6.	Lines 388-389 – The statement “Other notable genes were CACNA1C and DST.” is unclear. DST is already mentioned on line 385. Four target genes for the 13 miRNA with 7 target sites are given. What is notable and different about these two genes? What is different about DST mentioned here compared to line 385?**

We agree with your suggestion and have removed lines 388-389 as they do not provide novel conclusions.

REVIEWER 4

**1. The abbreviation should be spelled out for the first time use, e.g. What’s TD in abstract?**

Based on your suggestion the abbreviation has been addressed.

**2.** **Please clarify how 80 participants were sampled. Are they all the patients collected in a certain period of time?**

The issues with sampling addressed by the reviewer have been addressed in Materials and Methods section of the manuscript (Paragraph 2)

**3. 26 children were selected for clinical observations. How were these children selected? Also, how were their characteristics compared to the remaining children?**

Clinical evaluation and the mentioned issues by the reviewer were thoroughly addressed in materials and methods and discussion sections of the manuscript. Concordance between child psychiatrist and CARS 2 has been added in Table 2.

**4. Please provide the reasoning or support to use z-score of 1.5 as an outlier threshold, which seems quite small. ?**

The reasoning behind using 1.5 Z score as the outlier threshold has been explained in materials and methods section (line: 237)

5**. Line 213 reads, “used to describe the dependency of the dependent outcome, the diagnosis in this case,..” please clarify the outcome, ASD vs DD, without considering TD? If so, it implies that the analysis of interest only focus on ASD and DD sample. Then it would be useful to present Table 2 stratified by diagnosis status rather than pooling them together. If not, then please be clear about your modeling.**

According to your remark we stratified Table 2 based on diagnosis status.

**6. Lines 282 and 283 talk about training set and the validation set. However, it doesn’t mention anything about different dataset in the statistical analysis section. Please provide the detail and define your training and testing datasets!**

Random separation of the training and validation sets was done and multiple iterations were performed. A smaller validation subset was selected only for non-ASD DD because their sample size was smaller than others. Based on your suggestion the whole process of validation has been more thoroughly explained within the materials and methods section of the manuscript.
