# Supplementary material for: Identification of developmental disorders including autism spectrum disorder using salivary miRNAs in children from Bosnia and Herzegovina
Source: PLoS One. 2020 Apr 30;15(4):e0232351. doi: 10.1371/journal.pone.0232351 (PMC7192422; doi:10.1371/journal.pone.0232351)
Supplement: Attachment — Submitted filename: Response to Reviewers.docx [file pone.0232351.s015.docx]

Dear Reviewers,

Thank you so much for your time and such thorough comments and suggestions. All of your suggestions and comments have been addressed and the manuscript has been updated accordingly. Answers to your comments and how they have been addressed, in detail, please find below.

Once more thank you.

Sincerely,

Authors

**REWIER 1**

**1) “We have selected the 14 miRNAs based on their performance in Hicks et al., 2016.”— It is still unclear how the 14 miRNAs were selected. The main point of Hicks et al’s paper is 27 miRNAs with overlap across ASD studies; however, I do not see much overlap with the 27 miRNAs discussed in that paper.**

There are 2 articles published in 2016 by Hicks and his colleagues. The article titled “Salivary miRNA profiles identify children with autism spectrum disorder, correlate with adaptive behavior, and implicate ASD candidate genes involved in neurodevelopment” has found 14 differentially expressed miRNAs between TD and ASD children. Hence, based on their predictive performance they were chosen for analysis within the B&H population.

**2) It seems the authors are not even familiar with the statistical techniques you used in the manuscript. Cross validation (CV) was used because there is no replication study; however, this key word was not even clearly written.**

The issue with clarifying the terminology regarding validation was addressed. The term cross validation is now used instead of using the term validation on its own (Line: 257-258; 392-394).

**REWIER 3**

**1) It seems that there may be confusion related to the training and validation sets, or at least the terminology used, which still may need clarification. Specifically, it appears as though the entire cohort was used as training less ~10% held back for validation. This was repeated through ‘multiple iterations’ (line 258) using the software. I would contend that this would generally be recognized as cross-validation and the paper should be clear at some point that validation is referring to cross validation, which is different from a fully withheld and independent validation set. Similar, a limitation outlining the need for a fully independent sample cohort for validation should be included.**

As suggested by the reviewer, we have addressed the issue with using the term validation without stating it is cross validation. In materials and methods, we have changed validation to cross validation and have made clear that any type of validation which is mentioned later in the manuscript should be considered as a cross validation (Line: 257-258).

As suggested by the reviewer, we have addressed the limitation of the study regarding the need for an independent sample cohort for validation (Line: 392-394)

**2) The change of using the term spit to expectorant/expectorate is improper as it implies collection of phlegm and mucus from the lungs or throat rather than saliva (which it is meant to be reflected). This should be changed back to the previous wording (lines 107, 152).**

As suggested by the reviewer, the wording in lines 107 and 152 was returned back to the previous wording and the word expectorate was replaced by saliva.

**3) Other corrections are required in the tables in S6, S8-S11 with respect to formatting and decimals.**

Formatting corrections were made in the tables mentioned by the reviewer.
